# Epidemiological Profile of Bacterial Infections in Burn Patients Over a Five-Year Period

**DOI:** 10.7759/cureus.74848

**Published:** 2024-11-30

**Authors:** Ahmed Ibrahim Youssouf, Elmostafa Benaissa, El Mehdi Belouad, Zakaria Malihy, Yassin Benlahlou, Adil Maleb, Mariama Chadli, Mostafa Elouennass

**Affiliations:** 1 Bacteriology Laboratory, Faculty of Medicine and Pharmacy, Mohammed V Military Training Hospital, Mohammed V University, Rabat, MAR

**Keywords:** bacterial infections, burns, gram-negative bacilli, gram-positive cocci, resistance

## Abstract

Introduction: Burn patients are highly susceptible to bacterial infections, which significantly increase morbidity and mortality. Destruction of skin barriers following burns creates an ideal environment for tissue colonization by pathogenic microorganisms.

Objectives: The aim of our study is to establish the epidemiological profile of bacterial infections in burn patients hospitalized in the Burns and Plastic Surgery Department of the Mohamed V Military Teaching Hospital (HMIMV) in Rabat and to describe their sensitivity to antibiotics.

Materials and methods: This retrospective study spanned five years, from October 1, 2017, to December 31, 2022. During this period, a total of 548 samples were obtained, of which 366 (66.78%) were positive, corresponding to isolates of 39 non-redundant bacterial strains identified in 188 patient samples. Among these patients, 123 (65.42%) were males and 65 (34.22%) were females, yielding a sex ratio of 1.89. The mean age of the patients was 47.1 years, with an age range of 9 to 89 years. Microbiological and demographic data were collected for analysis. Bacterial isolates were identified using conventional bacteriological methods, and their antibiotic susceptibility was tested according to EUCAST 2019 guidelines.

Results: Of 548 samples, 406 (74.08%) skin burns, 74 (13.5%) blood cultures, 29 (5.29%) urine samples, and 27 (4.92%) central catheter samples were analyzed. Among the gram-negative, non-fermenting bacilli (GNB) bacilli, *Pseudomonas sp.* (n=92; 16.78%) were in the majority, with *Pseudomonas aeruginosa* accounting for 85 (92.39%) cases, while *Acinetobacter sp.* represented 78 (14.23%) cases, including 74 (94.87%) of *Acinetobacter baumannii*. Gram-positive bacteria were dominated by *Staphylococcus sp. *(n=123; 22.44%), including *Staphylococcus aureus* (n=60; 49.18%) and coagulase-negative *Staphylococcus* (n=63; 51.63%). Antibiotic resistance rates were particularly high among gram-negative bacteria, with resistance to carbapenems and cephalosporins being particularly alarming in *Acinetobacter baumannii*. Among gram-positive cocci, strains of* Staphylococcus aureus *showed moderate resistance to several antibiotics but remained susceptible to glycopeptides.

Conclusion: Non-fermentable gram-negative bacilli, particularly *Pseudomonas* and *Acinetobacter *species, are widespread in burn patients and show worrying levels of resistance to many antibiotics. These results underline the importance of judicious antibiotic management and rigorous infection control practices in burn care units.

## Introduction

Bacterial infections are a major complication in burn patients and are responsible for a significant increase in morbidity and mortality in this vulnerable population. The destruction of skin barriers following burns encourages microorganisms to colonize weakened tissues [1].

These fragile patients are most often exposed to a hostile hospital environment composed of microbial flora whose nature and density vary according to the local ecology. This microbial variability, most often with multi-drug resistance, combined with immune failure, explains the diversity of pathogens incriminated in burn victims and the complexity of the therapeutic management of these patients. A great deal of research has been carried out to determine the prevalence of bacterial infections in burn units. The pathogenic bacteria most often isolated during infections in burn victims are *Pseudomonas aeruginosa *(*P. aeruginosa*), *Acinetobacter baumannii* (*A. baumannii*), *Staphylococcus aureus *(*S. aureus*), *Klebsiella pneumonia* (*K. pneumonia*)and various coliform bacilli [2,3]. Nevertheless, few publications address the epidemiological situation of burn infections in Moroccan hospitals.

Our study aims to establish the epidemiological profile of bacterial infections in burn patients hospitalized in the Burns and Plastic Surgery Department of the Mohamed V Military Teaching Hospital (HMIMV) in Rabat and to describe their antibiotic sensitivity.

## Materials and methods

This retrospective study spans five years, from October 1, 2017, to December 31, 2022. During this period, a total of 548 samples were obtained, of which 366 (66.78%) were positive, corresponding to isolates of 39 non-redundant bacterial strains identified in 188 patients. The samples were obtained from the patients admitted to the burns and plastic surgery department and processed at the bacteriology laboratory of the Mohamed V Military Teaching Hospital (HMIMV) in Rabat. We included systematic samples including blood, urine, sputum, and tip of central line catheter collected from all patients admitted to the burns and plastic surgery department of the HMIMV who presented with symptoms of local (at the wound site) or systemic infection. We excluded samples from the same patients with the same isolate in different sites and the same antimicrobial susceptibility to avoid bias and violation of independence.

Diagnostic samples were processed using conventional bacteriological methods. Bacterial identification was performed based on morphological, cultural, and biochemical characteristics using API20E®, APINE®, and API STAPH® strips (BioMérieux, Lyon, France). Susceptibility testing was carried out on agar media and interpreted in accordance with the recommendations of the Antibiogram Committee of the French Microbiology Society/European Committee on Antimicrobial Susceptibility Testing (CASFM/EUCAST) [4].

The data are collected from the HMIMV bacteriology laboratory information system (SIL) concerning diagnostic samples from the burns and plastic surgery department received at the laboratory from October 2017 to December 2022. These data include: patient number, department, age, sex, nature of the samples, isolated germs, their resistance profiles (antibiogram data), and all the data obtained (data entry, data analysis, and results tables) were collected from the collection sheets and organized in Microsoft Excel 2016 (Microsoft Corp., Redmond, WA) for statistical analysis.

Descriptive and analytical statistics were performed using Jamovi software version 2.6.13 (https://www.jamovi.org). Qualitative variables are presented as relative frequencies (%) and absolute numbers (n), while quantitative variables are described by means and standard deviations or medians and interquartile ranges, as appropriate.

This study was conducted in strict adherence to the fundamental principles of medical research, including the principles of benefiting research, ensuring safety, and maintaining confidentiality.

## Results

Age

**Figure 1:**
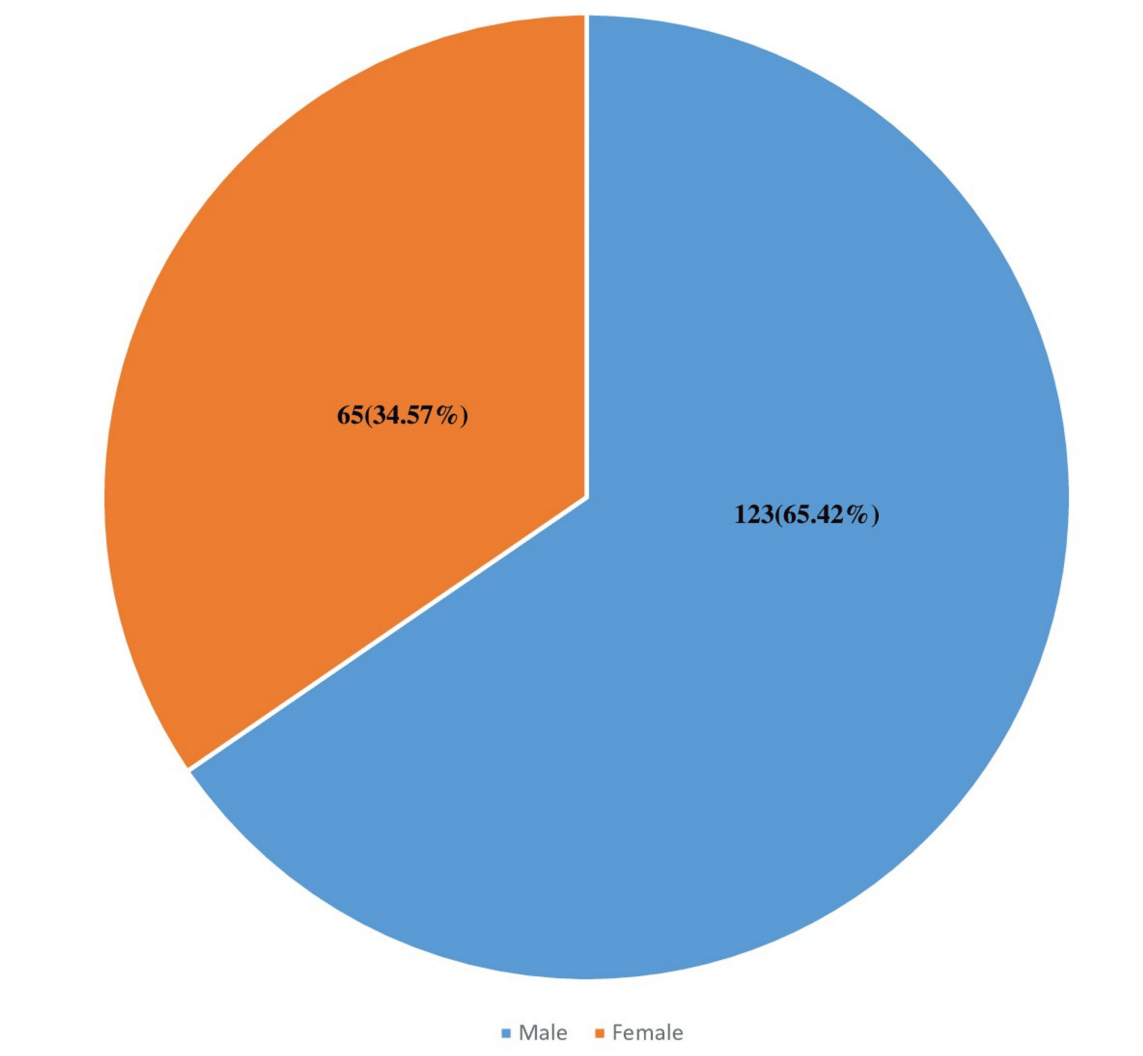
Gender distribution of patients

Over the course of the study, 548 samples were processed, with 366 (66.78%) samples being positive and 282 (33.21%) negative cultures. Our study involved 188 patients, 123 (65.42%) of whom were men and 65 (34.57%) women, with a male/female sex ratio of 1.89 (Figure 1). The mean age of the patients was 47.1 years ± 20.04, with an age range from 9-89 years. A total of 39 strains were isolated.

Distribution of sample and bacterial isolates

Of the 548 samples taken, 406 (74.08%) were from burns, of which 334 (60.94%) were swab samples. Around 60.94% were obtained by swabbing, 54 (9.85%) by aspiration of purulent collections, and 18 (3.28%) by skin biopsy. The bacteria most frequently found in these samples were *Staphylococcus sp.* with 105 (19.16%) isolates, followed by *Pseudomonas* *sp. *with 74 (13.5%) isolates, *Acinetobacter sp. *with 40 (7.29%) isolates, and *Proteus sp.* with 34 (6.2%) isolates.

Blood cultures yielded 74 bacterial strains (13.5%), of which *Acinetobacter sp.* was the most frequent with 20 (3.64%) isolates. *Staphylococcus sp.* was found in 12 (2.18%) cases, and *Enterococcus sp. *in 11 (2%) cases. Central line samples revealed 27 bacterial strains (4.92%), mainly *Acinetobacter sp.* with eight (1.45%) isolates and *Staphylococcus sp. *with five (0.91%) isolates. Urine analyses revealed 29 (5.29%) isolates, with a notable prevalence of *Escherichia sp.* in eight (1.45%) cases and *Pseudomonas sp.* in seven (1.27%) cases. Finally, respiratory samples identified 12 isolates (2.18%), dominated by *Acinetobacter sp.* with six (1.09%) cases followed by *Pseudomonas sp.* in two (0.36%) cases and *Haemophilus sp.* in one (0.18%) case (Table 1). The distribution of bacterial isolates was dominated by gram-negative bacteria in 334 (60.94%) while gram-positive bacteria represented only 214 (39.05%).

**Table 1:** Main sites of bacteriological sampling in burn victims during the study period *Cytobacteriological examination of urine, bladder catheter culture **Protected tracheal sampling, bronchoalveolar lavage, cytobacteriological examination of sputum

Sample site	Number of strains	Percentage
Skin sample	406	74.08%
Blood cultures	74	13.5%
Central catheter	27	4.92%
Urine sample *	29	5.29%
Respiratory sample **	12	2.18%

Detailed analysis of gram-negative bacteria

Gram-negative bacilli (GNB) were dominated by *non-fermenting *gram-negative bacilli (n=173; 31.56%) and Enterobacteriaceae (n=161; 29.37%). Concerning non-fermenting bacilli, *Pseudomonas sp.* (n=92; 16.78%) were in the majority with *P. aeruginosa* representing 85 (92.39%) of cases while *Acinetobacter sp.* represented 78 (14.23%) of the cases including 74 (94.87%) *A. baumannii*. Additionally, there were three (0.54%) isolates of *Stenotrophomonas maltophilia (S. maltophilia)*. In the Enterobacteriaceae family, there were 46 (8.39%) *K. pneumonia*, 37 (6.75%) *Proteus sp.*, 36 (6.56%), *Enterobacter sp.*, 27 (4.92%) *Escherichia coli (E.coli)*, eight (1.45%) *Morganella sp.*, three (0.54%) *Providencia sp.*, two (0.36%) *Serratia sp.*, one (0.18%)* Citrobacter sp.*, and one (0.18%) *Haemophilus influenzae*.

Detailed analysis of the profile of gram-positive bacteria

**Figure 2:**
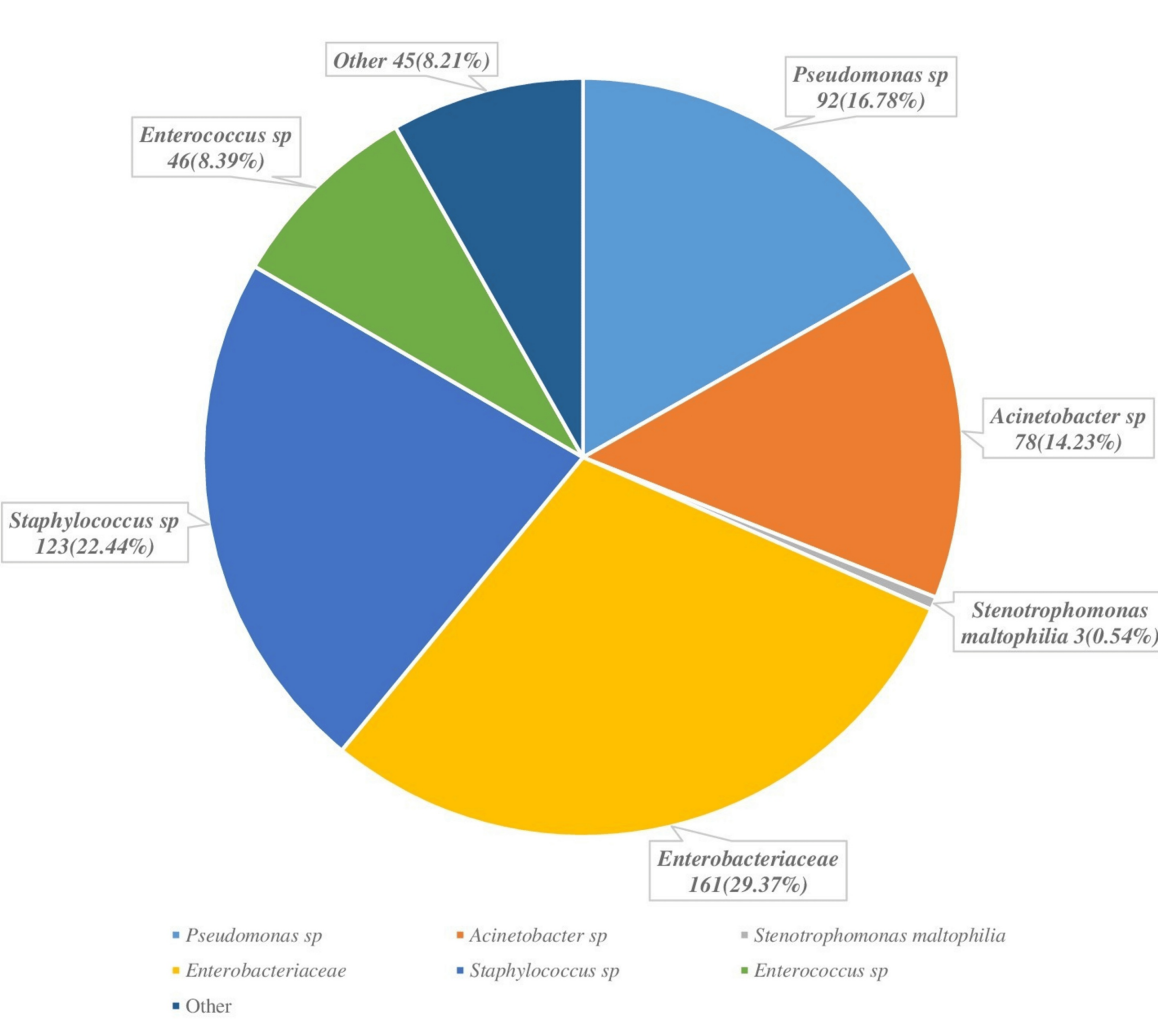
Bacterial distribution and isolates (n=548)

Gram-positive bacteria were dominated by *Staphylococcus sp.* 123 (22.44%), including *S.aureus* (n=60; 49.18%) and coagulase-negative* Staphylococcus *(n=63; 51.63%), followed by *Enterococcus sp.* (n=46; 8.39%) including *Enterococcus faecalis *(n=33; 71.73%) and *Enterococcus faecium *(n=13; 28.26% ). The other species were represented by *Corynebacterium sp.* (n=21; 3.83%), *Streptococcus sp.* (n=17; 3.1%), *Bacillus sp.* (n=4; 0.72%) and* Leuconostoc sp.* (n=3; 0.54%) (Figure 2).

Susceptibility to antibiotics

**Figure 3:**
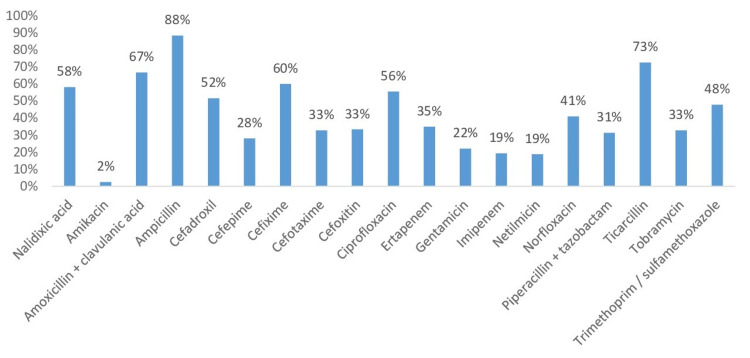
Antibiotic resistance profile of Enterobacterial isolates

The antibiotic susceptibility profile of Enterobacteriaceae shows significant resistance to several commonly used antibiotics, including ampicillin (88%) and ticarcillin (73%), suggesting that these are largely ineffective against the strains tested. Moderate resistance is also observed for nalidixic acid (58%) and cefotaxime (56%). However, lower resistance rates to amikacin (2%), imipenem (19%), and netilmicin (19%) indicate that these may still be effective treatment options (Figure 3).

**Figure 4:**
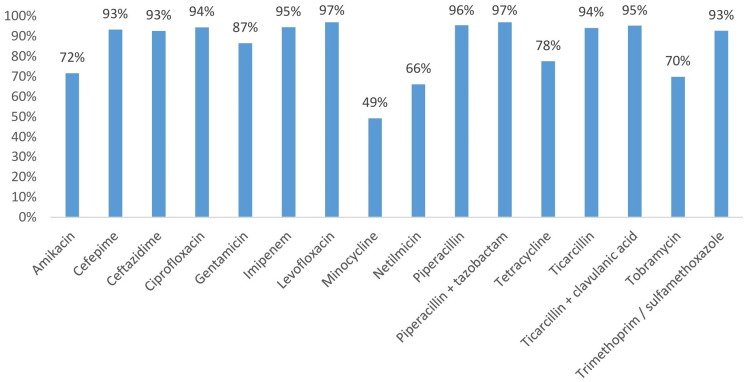
Antibiotic resistance profile of Acinetobacter baumannii isolates

The antibiotic susceptibility profile of *A. baumannii*, as illustrated in the graph, highlights a concerning level of resistance to several commonly used antibiotics. High resistance rates are observed for cefepime (93%), ceftazidime (93%), ciprofloxacin (94%), and gentamicin (87%), indicating limited efficacy of these antibiotics against the strain tested. Notably, imipenem and levofloxacin also show substantial resistance rates of 95% and 97%, respectively, suggesting significant challenges in treating infections with carbapenem-class antibiotics (Figure 4).

**Figure 5:**
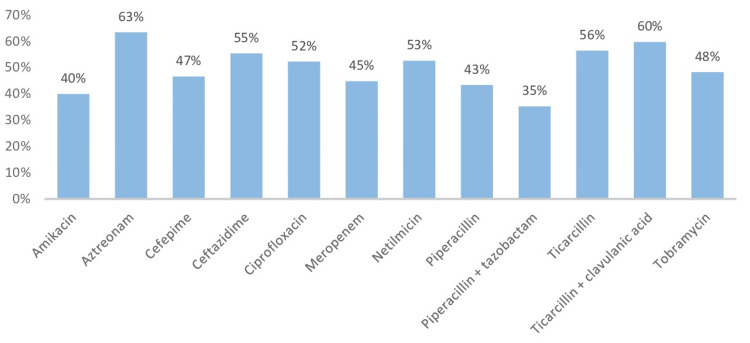
Antibiotic resistance profile of Pseudomonas aeruginosa isolates

Antibiotic susceptibility testing of *P. aeruginosa* showed notable resistance rates to several antibiotics: 63% for aztreonam, 55% for ceftazidime, 45% for meropenem, 40% for amikacin, 53% for netilmicin, and 48% for tobramycin. In addition, resistance to the quinolone ciprofloxacin was observed in 52% of isolates (Figure 5).

**Figure 6:**
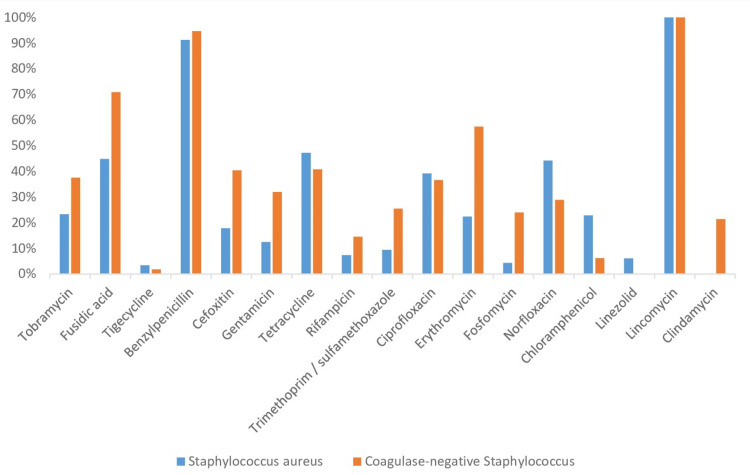
Antibiotic resistance profile of Staphylococcus aureus and Coagulase-Negative Staphylococci isolates

The antibiotic susceptibility profile of *S. aureus* and coagulase-negativeStaphylococci shows notable differences in resistance patterns. *S. aureus* shows high resistance to benzylpenicillin (91%) and erythromycin (39%) but lower resistance to commonly used drugs such as linezolid (0%) and clindamycin (0%). Conversely, coagulase-negative *Staphylococci* show even higher resistance to benzylpenicillin (95%), fusidic acid (71%), and erythromycin (57%), while showing no resistance to linezolid (0%) or lincosamides such as lincomycin (0%). Both groups retained a low level of resistance to gentamicin and trimethoprim/sulfamethoxazole (Figure 6).

**Figure 7:**
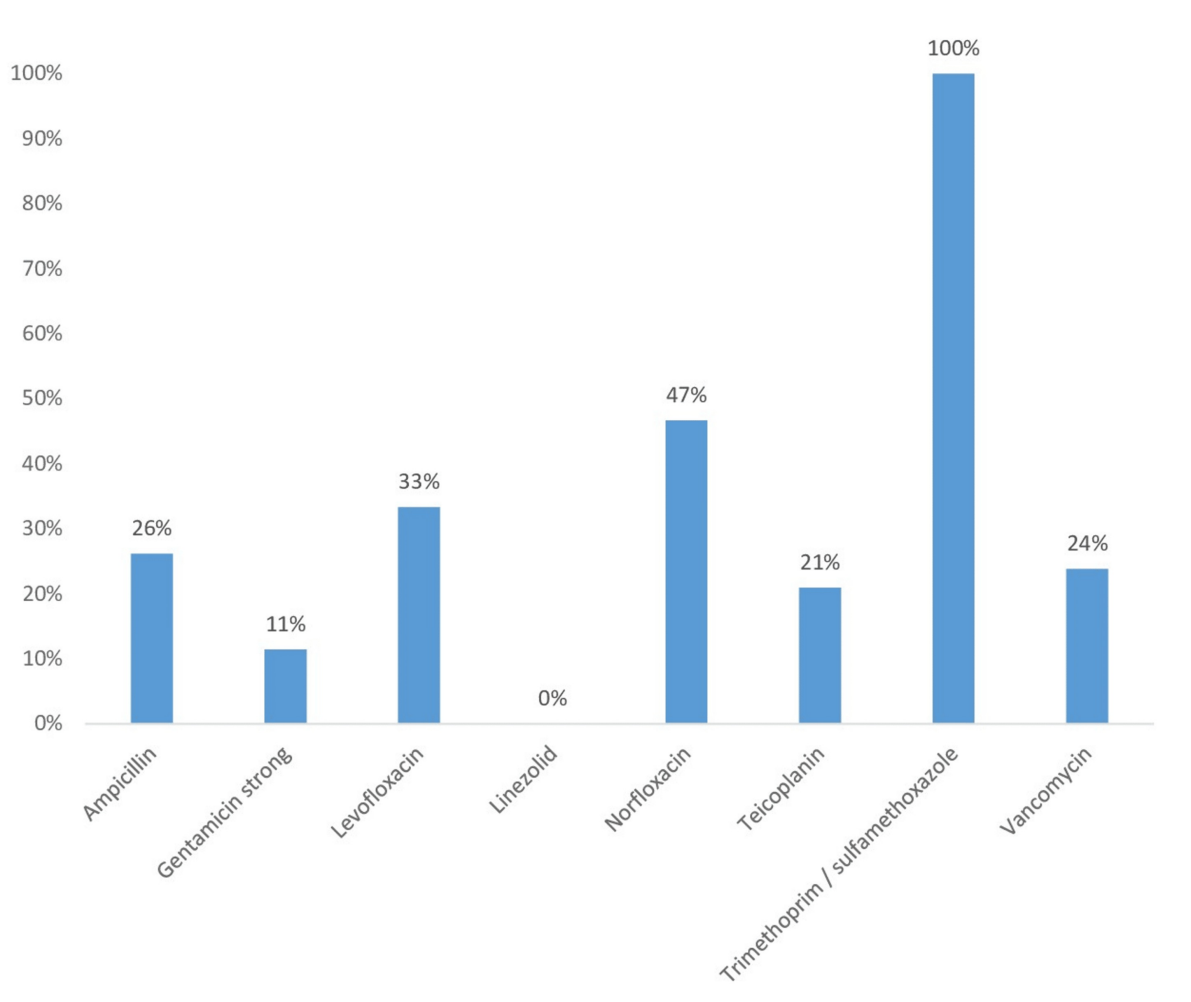
Antibiotic resistance profile of Enterococcus isolates

The antibiotic susceptibility profile of *Enterococcus* shows variable levels of resistance to the antibiotics tested. The strain shows high resistance to trimethoprim/sulfamethoxazole (100%) and moderate resistance to norfloxacin (47%) and levofloxacin (33%), suggesting limited efficacy of these agents. Conversely, linezolid shows complete susceptibility (0% resistance), indicating its potential as an effective therapeutic option for *Enterococcus* infections. In addition, low levels of resistance are observed for gentamicin (11%) and teicoplanin (21%), making them possible alternatives. Resistance to vancomycin (24%) is of concern, as vancomycin-resistant *Enterococci* pose significant therapeutic challenges (Figure 7).

## Discussion

During the study period, we were able to gather data on 188 patients hospitalized for burns with a male-female sex ratio of 1.89. This ratio correlates with the data in the literature, which presents a male predominance with values ranging from 1.44 to 2.55 [5]. This ratio can be attributed to men's tendency to engage in risky behaviors, as highlighted in the study by Essayagh et al. [6], as well as their higher prevalence in hazardous occupations [5]. The average age of our population is 47.1 years, ranging from 9-89 years. This value is close to the data encountered in the literature and fluctuates between 15.8 and 48.2 years [5]. Lionelli's study of 201 burn patients showed that age over 75 years, adjusted for burned body surface area (BSA), and the presence of infection are prognostic factors for mortality [7].

Our study of 548 bacterial isolates highlights a high prevalence of bacterial infections in burn patients, which is consistent with other similar studies [8]. Results by type of sample showed that skin samples were the most frequent (74.08%), followed by blood cultures, urine samples, and central catheters, which accounted for 13.5%, 5.29%, and 4.92% respectively. A study published in 2014 reported that the burns unit of the Mohamed V Military Training Hospital (HMIMV) had a higher prevalence of bacterial infection than in our study (on 112 samples taken, 76.8% were positive). This study showed that blood cultures were the most frequent samples taken (36%), followed by skin swabs (32%), urine (10%), and central catheters (13.4%) [6]. Another retrospective study carried out over a four-year period (2007-2010) at the HMIMV showed that positive swabs from burn wounds were the most frequent (48.9%), followed by positive blood cultures (22.1%), urine (16.3%) and positive catheters (2%) [6,9]. Furthermore, a study by Ekrami et al. conducted in a burns center in Iran also indicated that positive swabs from burn wounds were the most frequent (76.9%), followed by blood cultures (18.6%) and urine (8.9%) [10,11].

In our study, non-fermentative GNB (31.56%) were the majority, with *P. aeruginosa *and *A. baumannii* isolated in 92.39% and 94.87% of cases, respectively. For gram-positive cocci, *S. aureus* and coagulase-negative *Staphylococcus* were predominant in 49.18% and 51.63% of cases. Finally, Enterobacteriae*,* such as *K. pneumoniae* and *Proteus mirabilis*, were isolated in 8.39% and 6.56% of cases, respectively.

These results are similar to those of the study carried out in 2011 at the Military Hospital of Rabat which reports the abundance of non-fermenting gram-negative bacilli (39.7%), followed byEnterobacteriae (35.7%) and gram-positive cocci (24.6%) [9]. *P. aeruginosa *was the predominant species, also characterized by its multi-resistance to antibiotics. This observation is corroborated by other studies by Kaushik et al. and Revathi et al. [12,13]. Notably, the resistance rate is higher compared to that from 2014 in the same unit (Military Hospital of Rabat) where resistance to ticarcillin, piperacillin, ceftazidime, and imipenem were 52.6%, 26.3%, 26.3%, and 36.8%, respectively [6].

In this regard, a study carried out at the Center for Traumatology and Major Burns in Tunisia (2013) reported that the resistance rates of *Pseudomonas* to piperacillin, ceftazidime, imipenem, and gentamicin were respectively 23%, 24.6%, 31.1% and 44.1% [14]. *A. baumannii *is among the most opportunistic pathogens causing infection in burn centers and poses a treatment problem due to its ability to acquire resistance factors [15]. It should be noted that the remarkable frequency of *A. baumannii*, the second most isolated species in our study, is explained by its persistence in the environment and its hand-to-hand transmission.

Our observations concerning *S. aureus* show high resistance to benzylpenicillin (91%) but a total sensitivity to linezolid and clindamycin. This is consistent with the results of other studies where linezolid remained effective against MRSA strains [7] and had a resistance rate significantly lower than those of a study carried out at the Ibn Rochd University Hospital, which found the following resistances: oxacillin 46.2%, gentamicin 53.8%, erythromycin 46.2%, and lincomycin 7.6% [11]. They are also lower than those of a study published in 2013 in Douala, Cameroon, which revealed a methicillin resistance of 55.2% [16], close to that observed by the study carried out by Siah et al. (58.1%) [15]. The majority of the strains of *S. aureus* identified also showed sensitivity to glycopeptides. However, the emergence of strains of *S. aureus* with reduced sensitivity to Vancomycin constitutes a major problem, especially since the first Japanese report describing the isolation of a strain of *S. aureus* resistant to vancomycin [17].

Regarding coagulase-negative *Staphylococci,* our results are higher than those reported by the study conducted at the bacteriology laboratory of the Douala General Hospital, which was respectively 72%, 33%, and 46.1% for methicillin, gentamicin, and erythromycin. No strains resistant to glycopeptides were found [16]. Our results are also significantly higher than those of the study conducted at the Ibn Rochd University Hospital, which found low resistance rates: oxacillin (11.1%) and gentamicin (11.1%) [11]. In addition, *Enterococcus *species, identified in 8.39% of our isolates, are comparable to the reported results that note their presence in burn wounds, although generally at lower frequencies compared to *Staphylococcus* and *Pseudomonas* species [18].

Limitations of the study include its single-centre, retrospective design, which may limit generalizability. Additionally, the microbial flora of this Moroccan hospital may differ from that of other settings due to regional factors affecting bacterial colonization, such as climate and local antibiotic practices. Future studies should explore the genetic basis of resistance in these isolates and evaluate the efficacy of alternative treatment protocols, such as combination therapy and novel antimicrobials.

## Conclusions

This study sheds essential light on the microbial profile and antibiotic resistance patterns in burn patients at the Mohamed V Military Teaching Hospital in Rabat, Morocco. The results highlight a high prevalence of gram-negative bacteria, particularly *P. aeruginosa* and *A. baumannii*, which are associated with severe burn cases. The significant resistance observed in these pathogens to commonly administered antibiotics, including carbapenems, highlights the challenges of managing burn wound infections. Furthermore, the persistence of *S. aureus *and *Enterococcus *species among gram-positive isolates highlights the complexity of microbial colonization in burn wounds, where initial colonizers can subsequently be outcompeted by more resistant organisms. Our results encourage clinicians to use anti-infectives rationally and to reinforce hygiene measures in these patients in order to decrease the prevalence of these infections and, subsequently, the resulting morbidity and mortality.
